# Analysis of recanalization of deep venous thrombosis: a comparative study of patients treated with warfarin vs. rivaroxaban

**DOI:** 10.1590/1677-5449.180111

**Published:** 2019-06-17

**Authors:** Polyana Klomfass Piati, Aline Krampe Peres, Danielle Oliveira de Andrade, Mirela Andressa Jorge, Jeferson Freitas Toregeani

**Affiliations:** 1 Faculdade Assis Gurgacz – FAG, Cascavel, PR, Brasil.

**Keywords:** venous thrombosis, anticoagulant, ultrasonography doppler, Rivaroxaban, Warfarin

## Abstract

**Background:**

Deep venous thrombosis (DVT) strikes around ten million people worldwide every year and is associated with major complications including pulmonary embolism and post-thrombotic syndrome. Anticoagulation is the standard treatment, with administration of heparins, vitamin K antagonists, fondaparinux, or, more recently, direct oral anticoagulants (DOACs). Anticoagulants reduce thrombus progression and facilitate natural thrombolytic mechanisms, leading to a phenomenon known as recanalization, which can occur in varying degrees and over variable periods of time, under influence from many different factors, including the type of anticoagulation employed.

**Objectives:**

To evaluate the degree of recanalization and the time taken, by analysis of color Doppler ultrasonography (CDU) reports from patients with DVT treated with DOACs or with heparin + warfarin.

**Methods:**

A retrospective analysis was conducted of demographic data and CDU reports from patients with DVT who had been treated from January 2009 to December 2016. These patients were classified into two groups, according to the treatment given: Group I (heparin + warfarin): 26 patients; or Group II (rivaroxaban): 51 patients. The primary outcomes assessed were degree of recanalization and time taken.

**Results:**

Recanalization rates at 30, 90, and 180 days were 10%, 52.5%, and 78.9%, respectively, in Group I, and 55.3%, 83.5%, and 92.4%, respectively, in Group II, with statistically significant difference (p = 0.041).

**Conclusions:**

Both treatments led to recanalization. Recanalization occurred earlier among patients treated with rivaroxaban.

## INTRODUCTION

Deep venous thrombosis (DVT) is characterized by acute formation of blood clots in deep veins and can cause partial or total obstruction of the venous lumen. It is included in the nosological entity venous thromboembolism (VTE), which encompasses both DVT and pulmonary embolism (PE).^1^

Venous thromboembolism occurs in two out of every 1,000 individuals each year, with a rate of recurrence of 25%,^2^ while incidence increases to as much as 7 per 1,000 among people over the age of 70 years.^3^ It is considered an important cause of morbidity and mortality and requires early diagnosis, because of the risk of progression to PE,^4^ which has a mortality rate in the range of 5 to 15%,^2^ and to other morbidities such as chronic venous insufficiency and chronic pulmonary hypertension.^5^

One controversial element in the natural history of DVT is its progression. After an episode of DVT, an acute inflammatory response occurs in the vein wall and in the thrombus, leading to a dynamic process of thrombus regression by recanalization.^1^ This process involves fibrocellular organization of the thrombus, which includes contraction of the thrombus, formation of multiple cracks between thrombus and tunica intima, local fibrinolysis, and fragmentation of the thrombus after cellular invasion by neoformed vessels.^6^

Presence of thrombi and the recanalization process can damage venous valves, giving rise to valve incompetence. This condition, or persistent obstruction of the vein by residual thrombus, or even both, can cause chronic venous hypertension, causing post-thrombotic syndrome (PTS).^7^ The factors associated with risk of development of PTS of greater or lesser severity vary, and it is difficult to identify patients who are more prone to PTS.^8^ The probability of patients with idiopathic DVT developing PTS is in the range of 20 to 50%.^9^

Unfractionated heparin (UFH) was discovered in 1916 and became the first choice treatment for DVT during the 1930s. Soon after, the vitamin K antagonists (VKA) warfarin and phenprocoumon were developed. Low molecular weight heparins (LMWH) emerged during the 1980s.^1^ Up until 2011, the majority of patients were treated using regimens that followed VTE prophylaxis and treatment guidelines that predate the ninth edition of the American College of Chest Physicians’ (ACCP) Evidence–Based Clinical Practice Guidelines. In general, treatment of DVT is maintained for around 6 months, depending on patient progress and the thrombus site.^10^

In the 2000s, direct oral anticoagulants (DOACs) began to be released, offering as primary benefits freedom from laboratory monitoring, oral posology and a wider therapeutic window. Rivaroxaban and apixaban are employed without initial parenteral anticoagulation, in contrast with dabigatran and the more recent edoxaban, which need initial parenteral anticoagulation.^1^^,^ 11

Introduction of DOACs into routine clinical practice was supported by the results of many controlled clinical trials, meta-analyses, and real-life studies with large numbers of patients.^11^ Several different studies have shown that treatment with DOACs is not inferior to treatment with warfarin in terms of efficacy and safety. Examples of such studies include RE-COVER for dabigatran, EINSTEIN for rivaroxaban, AMPLIFY for apixaban, and HOKUSAI for edoxaban. These studies confirmed that treatment with DOACs was not inferior to warfarin, with similar or superior safety to the standard parenteral anticoagulation regimen followed by oral warfarin.^11^^,^ 12

Some reports have demonstrated that DOACs have the capacity to stimulate regression of thrombi, whether in the venous system or in other locations, such as the cardiac chambers.^13^^,^ 14 However, with regard to recanalization, few studies have compared ultrasonographic findings from patients treated with DOACs or warfarin.

The objective of this study was to assess the degree of recanalization and the time taken for recanalization by analysis of color Doppler ultrasonography (CDU) reports from patients with DVT treated either with rivaroxaban monotherapy or with parenteral heparin followed by oral warfarin.

## MATERIALS AND METHODS

The research on which this article is based was conducted in full compliance with Brazil’s National Health Council (Conselho Nacional de Saúde) Resolution 466/12 and was submitted to the Research Ethics Committee at the Centro Universitário, Faculdade Assis Gurgacz and approved under certificate number 51793015.0.0000.5219.

Reports from serial CDU examinations of 77 DVT patients treated from January 2009 to December 2016 were reviewed. Patients were classified into two groups according to the treatment received: Group I – heparin + warfarin: 26 patients (33.76%); or Group II – rivaroxaban: 51 patients (66.24%). All CDU examinations were performed by the same team of ultrasonographers, who described segments with thrombi using the traditional anatomic nomenclature: common iliac vein, external iliac vein, common femoral vein, deep femoral vein, femoral vein, popliteal vein, anterior and posterior tibial veins, fibular veins, and muscular veins. The baseline study variable was the sum of involved segments. As recanalization progressed, recanalized segments were excluded from the counts, leaving the number of segments still affected by thrombi at the end of the follow-up period. These recanalization data were also stratified in terms of percentage recanalization and time taken for recanalization.

The inclusion criteria were all patients with DVT confirmed by CDU for whom at least three follow-up vascular ultrasound examinations were available. From almost 500 CDU examinations performed during the period to evaluate DVT, serial examinations for 77 patients who fulfilled all criteria were selected. The exclusion criteria were patients for whom only two vascular ultrasound reports were available, and patients who had not been treated with either of the two classes of medications assessed in this study.

## RESULTS

Forty-two (54.55%) of the 77 patients studied were male. Mean age was 59.03±15.98 years. There was no statistically significant difference between the groups in terms of demographic profile (p = 0.42). The mean international normalized ratio (INR) for patients in Group I was 2.71±0.82.

At study outset, patients in Group I had a mean of 3.11 segments involved with thrombi. The patients in Group II had a mean of 2.76 involved segments. At the end of treatment, the number of segments still involved (with partial recanalization or no recanalization) was a mean of 0.38 in Group I and 0.14 in Group II, with no statistically significant difference between groups (p = 0.13).

In terms of percentage recanalization, patients in Group II exhibited quicker recanalization during the initial months of treatment and, when partial and total recanalizations were compared, there was a statistically significant difference in favor of patients treated with rivaroxaban (p = 0.041). Analyses of recanalization percentages in each group are shown in Table 1.

**Table 1:** Percentage total or partial recanalization, by duration of treatment.

**% cases recanalized after**	**Warfarin** **(Group I)**	**Rivaroxaban** **(Group II)**
30 days	10.00%	55.33%
90 days	52.50%	83.46%
180 days	78.88%	92.39%

p = 0.041.

## DISCUSSION

In this study, the majority of patients treated up to 2011 were given conventional treatment with UFH + VKA or LMWH + VKA. All of these patients were treated in hospital, with a minimum stay of 4 days. Inclusion of DOACs in clinical practice from 2011 onwards enabled significant changes to the way patients with VTE were treated. Since then, many patients with VTE have been treated with DOAC monotherapy at our service. Electronic patient records have been available at our institution since 2009, so we chose that start date for data collection.

Warfarin has been used in day-to-day clinical practice since the 1950s, so there is a wealth of information on its effects and limitations. One negative aspect of warfarin is its slow onset of action, because its effects are the result of interference in cyclic conversion of vitamin K and its 2.3-epóxido, blocking synthesis of the coagulation factors that are dependent on it (factors II, VII, IX, and X). Its anticoagulant effects do not therefore occur until factors already present in the circulation have been metabolized, which typically takes 36-72 h.^15^ Additionally, it has slow clearance, a narrow therapeutic window, and a large number of drug interactions, which means that it is necessary to continuously monitor activated partial thromboplastin time and INR and repeatedly adjust daily doses to achieve the ideal therapeutic dose.^16^

Rivaroxaban is an oral anticoagulant that acts as a reversible and specific factor Xa inhibitor. It has predictable pharmacokinetics and pharmacodynamics, with fewer interactions with foods, medications, and individual characteristics such as age, sex, weight, and ethnicity, in addition to low hepatic toxicity. One very positive factor is the wider therapeutic window than warfarin, which basically enables the same therapeutic result to be achieved despite physiological variations in the drug’s serum concentration. This may help to achieve better and faster recanalization of thrombi, because of reduced external interference in its anticoagulant effects.^17^

It is also necessary to take patient treatment adherence into account, since the simplicity of using a single drug to manage acute DVT from the initial phase with a higher dosage (30 mg) for 3 weeks, followed by a lower dose (20 mg) to maintain standard treatment, can potentially improve the risk/benefit profile of anticoagulation.^18^^,^ 19 In this case, compliance is improved by the posological convenience (a single daily dose for the majority of the treatment), the fact that laboratory testing to monitor activated partial thromboplastin time and INR is unnecessary, low incidence of side effects, and good tolerability.^20^ In the EINSTEIN-DVT and EINSTEIN PE studies, this regimen was not significantly inferior in terms of efficacy or safety when compared with the standard treatment with LMWH followed by VKA.^18^

However, it should not be forgotten that these new drugs also have their limitations, including the fact that they cannot be given to expectant mothers, breastfeeding mothers, patients with chronic advanced stage kidney disease, or patients with severe liver disease; the lack of a widely-available antidote; and the relatively high cost. These limitations create barriers to widespread use of these drugs for prophylaxis and treatment of VTE, particularly in Brazil.^21^

The ODIXa-DVT study^19^ reported results for the incidence of thrombus regression in patients treated with rivaroxaban after 21 days of treatment and compared different doses of the drug – 10 mg, 20 mg, 30 mg (2x/day) and 40 mg (1x/day), observing improvements in thrombotic load, measured as an increase ≥ 4 points in thrombus score, in 53%, 59.2, 56.9%, and 43.8% of patients respectively. The same study observed the same improvement in scores in 45% of patients treated with LMWH + VKA.

The J-EINSTEIN-DVT/PE randomized study, conducted in Japan, compared oral rivaroxaban in isolation (10 mg or 15 mg – 2x/day for 3 weeks followed by 15 mg/day for up to 12 months) with UFH+VKA in patients with DVT or PE. Imaging exams showed normalization after 22 days of treatment in 27% of the patients who were given rivaroxaban and in 15.8% of patients treated with UFH+VKA. At the end of the treatment, 62% of the patients in the rivaroxaban group and 31.6% in the UFH+VKA group exhibited normalization on examinations.^22^ In our study, examinations of patients after 30 days of treatment revealed partial or total recanalization rates of 10% and 55.3% in Groups I (heparin + warfarin) and II (rivaroxaban), respectively. At the end of the J-EINSTEIN-DVT/PE study, 95.8% of the patients in the rivaroxaban group exhibited improvement or normalization on examinations, compared to 89.5% of patients treated with UFH+VKA. In our study, after 180 days, 92.39% of the patients treated with rivaroxaban and 78.88% of the patients treated with LMWH + warfarin exhibited improvement or normalization on CDU.

As described above, many patients with DVT develop PTS. The residual thrombi load and the inflammatory process triggered by the thrombotic episode can cause irreversible sequelae. Development of PTS increases the average costs of DVT treatment by 74 to 81%.^23^ Therefore, early treatment and use of medications that have a full anticoagulant effect, in common with (the controversial) use of thrombolytic medication, appears to reduce the effects of PTS, impeding progression of the thrombus or, better still, helping to eliminate it more quickly.^24^ However, treatment with thrombolytics is associated with more hemorrhagic complications than treatment with conventional anticoagulant therapy (20% vs. 8%),^25^ and these complications include severe hemorrhages, such as, for example, intracranial bleeding (3.0% vs. 0.3%).^25^ Therefore, since treatment with anticoagulants is safer, it has been recommended for several years as first line of treatment for VTE in the majority of cases, and thrombolytics are only prescribed in specific cases.^10^

These factors should be considered in conjunction with the risk-benefit analysis of anticoagulant treatment and its efficacy for earlier regression of thrombi in VTE treatment, since insufficient anticoagulation can increase the risk of PTS and facilitate organization of the thrombus.^26^ Killewich et al.^27^ have reported evidence of lysis of thrombi and recanalization of venous segments in the first week after initial diagnosis. Mapping with CDU makes it possible to employ methods that quantify the recanalization process, such as those described by Porter et al.^28^ and Prandoni et al.^29^

Residual venous thrombosis has not yet been established as a marker for evaluation of duration of anticoagulation,^30^ and the current tendency is to maintain treatment for 3 months, with certain exceptions described in the latest ACCP consensus (2016).^31^ These guidelines state that treatment with anticoagulants for 3 months is effective for DVT episodes in patients without cancer (level 1B) and DOACs are preferred to warfarin (level 2B).^31^ This can also be seen in our study, in which 52.5% of the patients in Group I exhibited recanalization by 90 days, whereas in Group II, 83.46% already had echographic evidence of recanalization after the same period of time. At the end of the 180-day period, several patients in Group I were still showing progress in terms of recanalization, in contrast with Group II, in which there was little change from 90 to 180 days, since the majority of patients had already attained full recanalization and anticoagulant treatment had been withdrawn.

Earlier studies using repeated phlebographic examinations considered recanalization to be a later development, occurring over periods varying from 6 months to 1 years after the acute event.^32^ However, current reports from many different authors,^13^^,^ 14 and also our study, show that recanalization of thrombi in lower limb DVT in patients on DOACs can be faster than expected. In these cases, CDU is a valid and noninvasive tool, not only for initial diagnosis of DVT, but also for evaluation of the long term result, and can be used to guide initial patient management, providing information on adherence of the clot to the vein wall and on recanalization.^33^

Nevertheless, our study has several limitations, such as its single-center nature and the relatively small “n”, in addition to being retrospective. Despite these, the study is relevant because it offers an opportunity for theoretical reflections and analyses and guidance for future studies. More importantly, we must keep a space open for intense debate among health professionals on their experiences with DOACs and, at some point, on new drugs for treatment of VTE.

## CONCLUSIONS

Treatment of DVT with rivaroxaban or warfarin was effective and there was no difference in the number of segments that remained involved after 180 days’ follow-up of both groups. However, patients treated with rivaroxaban exhibited evidence of earlier recanalization than patients treated with warfarin.
