# Supplementary material for: Analysis of recanalization of deep venous thrombosis: a comparative study of patients treated with warfarin vs. rivaroxaban
Source: J Vasc Bras. 2019 Jun 17;18:e20180111. doi: 10.1590/1677-5449.180111 (PMC6636999; doi:10.1590/1677-5449.180111)
Supplement: Translation

# Translation: Análise do grau de recanalização da trombose venosa profunda: estudo comparativo de pacientes tratados com varfarina versus rivaroxabana

**Authors:** Polyana Klomfass Piati, Aline Krampe Peres, Danielle Oliveira de Andrade, Mirela Andressa Jorge, Jeferson Freitas Toregeani

**Affiliations:** 1 Faculdade Assis Gurgacz – FAG, Cascavel, PR, Brasil.

**Keywords:** trombose venosa, anticoagulante, ultrassonografia doppler, Rivaroxabana, Varfarina

## Abstract

**Contexto:**

A trombose venosa profunda (TVP) afeta anualmente cerca de dez milhões de pessoas no mundo e tem como principais complicações a embolia pulmonar e a síndrome pós-trombótica. O tratamento padrão é a anticoagulação, que pode ser realizada com heparinas, antagonistas da vitamina K, fondaparinux ou, mais recentemente, com anticoagulantes orais diretos (*direct oral anticoagulants*, DOACs). Os anticoagulantes diminuem a progressão do trombo e facilitam os mecanismos trombolíticos naturais, fato conhecido como recanalização, que pode ocorrer em graus e tempos variados, influenciados por diversos fatores, dentre eles o tipo de anticoagulação utilizado.

**Objetivos:**

Avaliar o grau e o tempo de recanalização através da análise de laudos de eco-Doppler colorido (EDC) de pacientes com TVP tratados com DOACs ou com heparina + varfarina.

**Métodos:**

Foram avaliados retrospectivamente os dados demográficos e os laudos dos EDC dos pacientes com TVP, tratados entre janeiro de 2009 a dezembro de 2016. Os pacientes foram divididos em dois grupos, de acordo com a terapêutica utilizada: Grupo I (heparina + varfarina): 26 pacientes; Grupo II (rivaroxabana): 51 pacientes. Os principais itens observados foram o grau e o tempo para a recanalização.

**Resultados:**

Foram observadas taxas de recanalização aos 30, 90 e 180 dias de 10%, 52,5% e 78,9%, respectivamente, no Grupo I, e de 55,3%, 83,5% e 92,4%, respectivamente, no Grupo II, com diferença estatisticamente significativa (p = 0,041).

**Conclusões:**

Ambos os tratamentos promoveram recanalização. Houve recanalização mais precoce no grupo de pacientes que utilizaram a rivaroxabana.

## INTRODUÇÃO

A trombose venosa profunda (TVP) caracteriza-se pela formação aguda de um coágulo em veias profundas, podendo levar à obstrução parcial ou total do lúmen venoso, e está compreendida dentro da entidade nosológica denominada tromboembolismo venoso (TEV), a qual engloba a TVP e a embolia pulmonar (EP)^1^.

O TEV pode ocorrer em dois para cada 1.000 indivíduos ao ano, com uma taxa de recorrência de 25%^2^, havendo um aumento da incidência para até 7 por 1.000 entre aqueles com idade superior a 70 anos^3^. É considerada uma causa importante de morbimortalidade e requer diagnóstico precoce, devido ao risco de evoluir para EP^4^, a qual apresenta uma taxa de mortalidade de 5 a 15%^2^, e para outras morbidades como insuficiência venosa crônica e hipertensão pulmonar crônica^5^.

Um dos aspectos controversos na história natural da TVP é a sua evolução. Após um episódio de TVP, uma resposta inflamatória aguda ocorre na parede da veia e no trombo, levando a um processo dinâmico de regressão do trombo por meio da recanalização^1^. Esse processo está envolvido na organização fibrocelular do trombo, que engloba a contração do trombo, a formação de fendas múltiplas entre o trombo e a camada íntima, a fibrinólise local e a fragmentação do trombo após a invasão celular por vasos neoformados^6^.

A presença do trombo e o processo de recanalização podem levar a danos nas válvulas venosas, originando assim a incompetência valvular. Essa condição, ou a obstrução persistente da veia pelo trombo residual, ou então ambas, causam hipertensão venosa crônica, acarretando a síndrome pós-trombótica (SPT)^7^. Os fatores associados ao risco de desenvolver SPT em maior ou menor grau são variados, sendo difícil identificar quais pacientes são mais propensos a desenvolvê-la^8^. Há uma probabilidade de cerca de 20 a 50% dos pacientes com TVP idiopática desenvolverem SPT^9^.

Descoberta em 1916, a heparina não fracionada (HNF) passou a ser a principal escolha para o tratamento da TVP a partir dos anos 30. Logo após, surgiram os antagonistas da vitamina K (AVK), representados pela varfarina e pela femprocumona. Nos anos 80, foi a vez das heparinas de baixo peso molecular (HBPM)^1^. Até 2011, a maioria dos pacientes utilizava o esquema de tratamento de acordo com as diretrizes de profilaxia e tratamento do TEV anteriores à nona edição do *American College of Chest Physicians Evidence – Based Clinical Practice Guideline* (ACCP). De modo geral, o tratamento da TVP se estendia por cerca de 6 meses, conforme a evolução do paciente e a localização do trombo^10^.

A partir dos anos 2000, surgiram os anticoagulantes orais diretos (*direct oral anticoagulants*, DOACs), cujos principais benefícios são a não necessidade de monitorização laboratorial, a posologia oral e a janela terapêutica maior. A rivaroxabana e a apixabana são utilizadas sem anticoagulante parenteral inicial, ao contrário da dabigatrana e mais recentemente da edoxabana, que necessitam de anticoagulante parenteral inicial^1^^,^ 11.

A introdução dos DOACs na prática clínica diária foi amparada em diversos ensaios clínicos controlados, metanálises e estudos de vida real com grande número de pacientes^11^. Vários estudos mostraram que a terapia com DOACs é não inferior à terapia com varfarina em termos de eficácia e segurança. São exemplos os estudos RE-COVER para dabigatrana, EINSTEIN para rivaroxabana, AMPLIFY para apixabana e HOKUSAI para edoxabana. Comprovou-se a partir desses estudos a não inferioridade do uso dos DOACs em relação à varfarina, com segurança similar ou superior ao esquema padrão de anticoagulante parenteral seguido do uso oral da varfarina^11^^,^ 12.

Alguns relatos têm demonstrado a capacidade dos DOACs em promover a regressão de trombos, seja no sistema venoso ou em outros locais, como nas câmaras cardíacas^13^^,^ 14 Entretanto, no caso da recanalização, estudos que compararam os achados ultrassonográficos de pacientes tratados com DOACs ou varfarina são escassos.

O objetivo deste trabalho foi avaliar o grau e o tempo de recanalização através da análise de laudos de eco-Doppler colorido (EDC) de pacientes com TVP tratados com monoterapia com rivaroxabana ou com heparina parenteral seguida de varfarina oral.

## MATERIAIS E MÉTODOS

A pesquisa que deu origem a este artigo foi realizada em concordância com a Resolução nº 466/12 do Conselho Nacional de Saúde e foi submetida ao Comitê de Ética em Pesquisa (CEP) do Centro Universitário, Faculdade Assis Gurgacz (FAG), com a respectiva aprovação sob CAE Nº 51793015.0.0000.5219.

Foram avaliados os laudos de EDC seriados de 77 pacientes portadores de TVP tratados entre janeiro de 2009 a dezembro de 2016. Os pacientes foram divididos em dois grupos de acordo com a terapêutica utilizada: Grupo I – heparina parenteral + varfarina: 26 pacientes (33,76%); Grupo II – rivaroxabana: 51 pacientes (66,24%). Os exames de EDC foram realizados pela mesma equipe de ultrassonografistas, que descreveram os segmentos acometidos pelos trombos seguindo a nomina anatômica tradicional: veia ilíaca comum, veia ilíaca externa, veia femoral comum, veia femoral profunda, veia femoral, veia poplítea, veias tibiais anteriores e posteriores, veias fibulares e veias musculares. A soma dos segmentos acometidos forneceu o dado de entrada no estudo. À medida que a recanalização foi acontecendo, foram excluídas da contagem os segmentos recanalizados, obtendo-se o número de segmentos ainda comprometidos por trombos ao final do período de avaliação. Além disso, os dados de recanalização foram estratificados quanto à porcentagem de recanalização e ao tempo em que ocorreram.

Os critérios de inclusão foram todos os pacientes com TVP confirmada por EDC e que possuíam pelo menos três exames de ultrassom vascular no acompanhamento. Dos quase 500 laudos de EDC realizados no período para avaliar TVP, foram selecionados os exames seriados dos 77 pacientes que preencheram todos os critérios. Os critérios de exclusão foram os pacientes com apenas um ou dois laudos de ultrassom vascular, bem como pacientes que não foram tratados com as duas classes de medicamentos avaliadas neste estudo.

## RESULTADOS

Dos 77 pacientes estudados, 42 (54,55%) eram do sexo masculino. A média de idade foi de 59,03±15,98 anos. Não houve diferença estatisticamente significativa entre os grupos quanto aos padrões demográficos (p = 0,42). A média da razão de normatização internacional (RNI) dos pacientes do Grupo I foi de 2,71±0,82.

No início do estudo, os pacientes do Grupo I apresentavam, em média, 3,11 segmentos comprometidos com trombos. Quanto aos pacientes do Grupo II, em média, 2,76 segmentos estavam comprometidos. Ao final do tratamento, a quantidade de segmentos ainda comprometidos (com recanalização parcial ou nenhuma recanalização) foi, em média, de 0,38 segmentos para o Grupo I e 0,14 segmentos para o Grupo II, não havendo significância estatística na diferença entre os grupos (p = 0,13).

No que se refere à porcentagem de recanalização, os pacientes do Grupo II apresentaram recanalização mais rápida nos meses iniciais do tratamento e, quando comparadas as recanalizações parciais e totais, houve diferença estatisticamente significativa favorável aos pacientes tratados com rivaroxabana (p = 0,041). As avaliações da porcentagem de recanalização em cada grupo estão representadas na Tabela 1.

**Tabela 1:** Porcentagem de recanalização total ou parcial em relação ao tempo de tratamento.

**% CASOS RECANALIZADOS EM**	**VARFARINA** **(GRUPO I)**	**RIVAROXABANA** **(GRUPO II)**
**30 DIAS**	10,00%	55,33%
**90 DIAS**	52,50%	83,46%
**180 DIAS**	78,88%	92,39%

p = 0,041.

## DISCUSSÃO

Neste trabalho, a maioria dos pacientes tratados até 2011 utilizavam a terapia convencional com HNF + AVK ou HBPM + AVK. Todos os pacientes foram tratados em regime de internação por pelo menos 4 dias. A inclusão dos DOACs na prática clínica a partir de 2011 promoveu mudanças significativas na forma de tratamento dos pacientes com TEV. Desde então, muitos pacientes com TEV têm sido tratados com monoterapia com DOAC em nosso serviço. Devido ao fato dos prontuários eletrônicos estarem disponíveis em nossa instituição a partir de 2009, utilizamos este período como inicial para coleta de dados.

A varfarina tem sido utilizada na prática clínica diária desde os anos 1950; portanto, existem informações abundantes sobre seus efeitos e também sobre suas limitações. Um ponto negativo da varfarina é o início de ação demorado, pois produz o seu efeito ao interferir na conversão cíclica da vitamina K e do seu 2,3-epóxido, bloqueando a síntese de fatores de coagulação dela dependentes (fatores II, VII, IX e X). Assim, o seu efeito anticoagulante não acontece até que os fatores já presentes na circulação sejam metabolizados, processo que leva tipicamente de 36-72 h^15^. Além disso, apresenta eliminação demorada, uma janela terapêutica estreita e uma grande gama de interações medicamentosas, o que demanda monitorização contínua do tempo de ativação da protrombina (TAP) e da RNI e mudanças repetidas da dose diária a fim de atingir a dose terapêutica ideal^16^.

A rivaroxabana é um anticoagulante oral que atua como inibidor reversível e específico do fator Xa. Apresenta farmacocinética e farmacodinâmica previsíveis, com menor interação com alimentos, medicamentos e características individuais como idade, sexo, peso e etnia, além de baixa toxicidade hepática. Um fator muito positivo é a janela terapêutica mais larga que a da varfarina, o que permite basicamente o mesmo resultado terapêutico mesmo que ocorram variações fisiológicas da concentração sérica da medicação. Isso pode auxiliar em uma melhor e mais rápida recanalização dos trombos, devido à menor interferência externa em seus efeitos anticoagulantes^17^.

É preciso levar em consideração a aderência do paciente ao tratamento, pois a abordagem simples de única droga para o tratamento agudo da TVP desde a fase inicial com dose mais alta (30 mg) por 3 semanas seguida da administração de dose inferior (20 mg) para continuidade de tratamento padrão potencialmente melhora o perfil do risco/benefício da anticoagulação^18^^,^ 19. Nesse caso, a adesão é favorecida pela comodidade posológica (dose única diária na maior parte do tratamento), não necessidade de monitorização laboratorial de TAP e RNI, baixa incidência de efeitos colaterais e boa tolerabilidade^20^. Nos estudos EINSTEIN-DVT e EINSTEIN PE, esse regime não demonstrou inferioridade em eficácia e segurança de maneira significativa quando comparado à terapia padrão com HBPM seguida de AVK^18^.

No entanto, não se deve esquecer que essas novas drogas também têm suas limitações, dentre as quais podemos citar a impossibilidade de serem utilizadas em gestantes, lactantes, pacientes renais crônicos em estágio avançado e pacientes com hepatopatia grave; a falta de antídoto amplamente disponível e o custo relativamente alto. Tais limitações impedem que esses novos medicamentos sejam amplamente usados para profilaxia e tratamento de TEV, principalmente no Brasil^21^.

O estudo ODIXa-DVT^19^ apresentou resultados sobre a incidência de regressão do trombo com uso de rivaroxabana aos 21 dias de tratamento e comparou dosagens diferentes do medicamento – 10 mg, 20 mg, 30 mg (2x/dia) e 40 mg (1x/dia) –, apresentando uma melhora na carga trombótica, demonstrada por um aumento ≥ 4 pontos do escore de trombo, em 53%, 59,2, 56,9% e 43,8% dos pacientes, respectivamente. No mesmo estudo, a mesma melhora do escore foi observada em 45% dos pacientes em uso de heparina + AVK.

O estudo randomizado J-EINSTEIN-DVT/PE, realizado no Japão, compararam a rivaroxabana oral isolada (10 mg ou 15 mg – 2x/dia por 3 semanas seguido de 15 mg/dia por até 12 meses) com HNF+AVK em pacientes com TVP ou EP. Houve normalização dos exames de imagem aos 22 dias de tratamento em 27% dos pacientes que utilizaram rivaroxabana e em 15,8% dos pacientes tratados com HNF+AVK. Ao final do tratamento, 62% e 31,6% dos pacientes dos grupos rivaroxabana e HNF+AVK apresentaram normalização dos exames, respectivamente^22^. Em nosso estudo, na avaliação dos pacientes após 30 dias de tratamento, foram encontradas taxas de recanalização parcial ou total de 10% e 55,3% nos Grupos I (heparina + varfarina) e II (rivaroxabana), respectivamente. Ao final do estudo J-EINSTEIN-DVT/PE, 95,8% dos pacientes do grupo rivaroxabana tiveram melhora ou normalização dos exames, enquanto que 89,5% dos pacientes tratados com HNF+AVK obtiveram melhora parcial ou total. Em nosso estudo, ao final de 180 dias, 92,39% dos pacientes tratados com rivaroxabana e 78,88% dos pacientes tratados com heparina + varfarina apresentaram melhora ou normalização do EDC.

Conforme descrito anteriormente, muitos pacientes com TVP evoluem para SPT. A carga de trombos residuais e o processo inflamatório gerado pelo episódio trombótico podem causar sequelas irreversíveis. O desenvolvimento da SPT aumenta o custo médio do tratamento da TVP em 74 a 81%^23^. Desse modo, o tratamento precoce e o uso de medicamentos que tenham um efeito anticoagulante pleno, bem como o uso discutível das medicações trombolíticas, parecem diminuir os efeitos da SPT, impedindo a progressão do trombo ou, melhor ainda, ajudando a eliminá-lo mais rápido^24^. No entanto, o tratamento com trombolíticos apresenta mais complicações hemorrágicas do que o tratamento com a terapia convencional com anticoagulantes (20% *versus* 8%)^25^, incluindo nessas complicações as hemorragias graves, como por exemplo, o sangramento intracraniano (3,0% *versus* 0,3%)^25^. Portanto, por apresentar maior segurança, a terapia com anticoagulantes vem sendo recomendada há vários anos como primeira opção no tratamento do TEV na maioria dos casos, e os trombolíticos são indicados somente em situações especiais^10^.

Esses fatores devem ser considerados em conjunto com a avaliação risco-benefício da terapia anticoagulante e a eficácia para a regressão de trombos mais precoce no tratamento do TEV, pois a anticoagulação insuficiente pode aumentar o risco de SPT e facilitar a organização do trombo^26^. Killewich et al.^27^ reportaram evidências de lise do trombo e recanalização de segmentos venosos já na primeira semana após o diagnóstico inicial. O mapeamento com EDC permite aplicar métodos que quantifiquem o processo de recanalização, como os descritos por Porter et al.^28^ e Prandoni et al.^29^.

A trombose venosa residual ainda não está estabelecida como um marcador para avaliar a duração da terapia anticoagulante^30^, sendo que atualmente há uma tendência ao tratamento por 3 meses, salvo exceções descritas no último consenso da ACCP (2016)^31^. Segundo este, o tratamento com anticoagulante pelo período de 3 meses para episódios de TVP em pacientes sem câncer tem se mostrado efetivo (grau 1B), tendo preferência o uso dos DOACs sobre a varfarina (grau 2B)^31^. Esse fato pode ser constatado em nosso trabalho, no qual se observou que, após 90 dias, 52,5% dos pacientes do Grupo I apresentavam recanalização, enquanto que, no Grupo II, 83,46% já tinham evidências ecográficas de recanalização. Ao final do período de 180 dias, vários pacientes do Grupo I continuaram a ter evolução no quesito recanalização, ao contrário do Grupo II, que teve pouca modificação entre 90 e 180 dias, tendo em vista que a maioria dos pacientes já tinha recanalização total, tendo inclusive terminado o tratamento com anticoagulante.

Os estudos prévios utilizando exames flebográficos repetidos consideravam a recanalização como uma reação tardia, ocorrendo em períodos que variavam de 6 meses até anos após o evento agudo^32^. Entretanto, os relatos atuais de diversos autores^13^^,^ 14 e o nosso estudo mostraram que a recanalização de um trombo nos membros inferiores com TVP em pacientes em uso de DOACs pode ser mais rápido do que o esperado. Nesses casos, o EDC representa uma ferramenta válida, não invasiva, não apenas para o diagnóstico inicial da TVP, mas também para avaliar o resultado a longo prazo, podendo orientar o manejo inicial do paciente e fornecendo informações sobre a fixação do coágulo na parede da veia e sobre a recanalização^33^.

Destarte, o estudo apresenta várias limitações, como concentrar-se em um único centro de tratamento e ter “n” relativamente pequeno, além do fato de ser retrospectivo. Apesar disso, o estudo é relevante, por possibilitar análises e reflexões para aprofundamento teórico e para novas pesquisas. Mais importante que isso, devemos manter aberto o espaço para intenso debate entre profissionais da saúde em relação às experiências com os DOACs e eventualmente com novas drogas para o tratamento do TEV.

## CONCLUSÃO

O tratamento da TVP com rivaroxabana ou varfarina foi efetivo, não havendo diferença na quantidade de segmentos que permaneceram comprometidos ao final de 180 dias de acompanhamento em ambos os grupos. No entanto, os pacientes tratados com rivaroxabana apresentaram evidências de recanalização mais precoce do que os pacientes tratados com varfarina.
